# Harmonization of SDQ and ASEBA Phenotypes: Measurement Variance Across Cohorts

**DOI:** 10.1007/s10862-025-10204-0

**Published:** 2025-03-07

**Authors:** Miljan Jović, Maryam Amir-Haeri, Kaili Rimfeld, Judith B. M. Ensink, Ramon J. L. Lindauer, Tanja G. M. Vrijkotte, Andrew Whitehouse, Stéphanie M. van den Berg

**Affiliations:** 1 Department of Learning, Data Analytics and Technology, Faculty of Behavioural, Management and Social Sciences, University of Twente, PO Box 217, Enschede, 7500 AE The Netherlands; 2 Social, Genetic and Developmental Psychiatry Centre, Institute of Psychiatry, Psychology & Neuroscience, King’s College London, London, UK; 3 Royal Holloway University of London, London, UK; 4 Location AMC, Department of Child and Adolescent Psychiatry, Amsterdam University Medical Center, Amsterdam, The Netherlands; 5 Location AMC, Department of Clinical Genetics, Amsterdam University Medical Center, Genome Diagnostics Laboratory, Amsterdam, The Netherlands; 6 Academic Centre for Child and Adolescent Psychiatry, Amsterdam, The Netherlands; 7 Location AMC, Department of Public and Occupational Health, Amsterdam University Medical Center, University of Amsterdam, Amsterdam, The Netherlands; 8 Amsterdam Public Health Research Institute, Amsterdam, The Netherlands; 9 Telethon Kids Institute, University of Western Australia, Perth, Australia

**Keywords:** Data harmonization, Measurement invariance, Anxiety, Depression, ADHD

## Abstract

Harmonizing the scores obtained by different instruments that measure the same construct enable researchers to combine them in one analysis. An important step in harmonization is checking whether there is measurement invariance across populations. This study aimed to examine whether the harmonized scores for anxiety/depression and ADHD obtained by two different instruments (the Child Behaviour Check List (CBCL) and the Strength and Difficulties Questionnaire (SDQ)) are measurement invariant across other countries, languages, and age groups. We used cohorts from Australia (1330 children aged 10–11.5 years), the Netherlands (943 children aged 11–13.5 years) and the United Kingdom (4504 children aged 14–19). We used the Bayesian method for modeling measurement non-invariance proposed by Verhagen and Fox, 2013a that we adapted for using on polytomous items and in a relatively small number of groups (cohorts). Results showed that there is hardly any differential functioning of harmonized anxiety/depression and ADHD scores obtained by CBCL and SDQ across cohorts. The same model that harmonizes measures in Australian 10-year-old children can also be used in cohorts from the UK and the Netherlands.

**Supplementary Information:**

The online version contains supplementary material available at 10.1007/s10862-025-10204-0.

## Introduction

In psychology and related fields, if we want to make comparisons between the test scores of individual participants and/or groups of participants who filled in different instruments that measure the same construct (e.g., anxiety) we have to harmonize the scores by putting them on a common scale. Without putting them on a common scale, we are not able to compare them. Making the scores obtained by different instruments comparable also enables researchers to pool data, which allows for more generalizability, larger sample size, and thereby statistical power of research results (Fortier et al., 2010; Fortier et al., 2011; Hamilton et al., 2011; Smith-Warner et al., 2006; Thompson, 2009; Van Den Berg et al., 2014). This is especially important in research consortia where different institutions use different instruments for the same construct, resulting in difficulties combining the data (Luningham et al., 2019; van den Berg et al., 2014). For example, for a large consortium, Jović et al. (2022) harmonized anxiety, depression, and ADHD scores measured with two widely used screening instruments for psychopathology in children: The Child Behaviour Checklist (CBCL) (Achenbach, 1991) and the Strengths and Difficulties Questionnaire (SDQ) (Goodman, 1997, 2001). Item Response Theory (IRT) was used to harmonize the scores and this increased the statistical power of the results compared to sum scores. The harmonization is conducted on a sample of participants from Australia.

It is not straightforward to find a common scale for a construct that is operationalized in different ways across questionnaires. Even more challenging is to find a common scale that is invariant^1^ across several cohorts, that have different languages, cultures and age groups. The success of harmonization in international studies depends on the extent that there is measurement invariance across different populations. Accordingly, an important step in the process of establishing a harmonized measure for a construct is to examine if harmonized measures posses measurement invariance. In other words, whether harmonized scores can be interpreted in the same way across countries, languages, and/or different age groups, and therefore usable for across-group comparisons or for increasing sample size and generalizability.

### Measurement Invariance

For measures to be useful for inter-individual comparisons, the relations between indicators of the measured construct (items) and the construct of interest must be equivalent across subgroups (Reise et al., 1993). This is particularly a concern when data comes from different countries with different languages and cultures, when items can be interpreted slightly differently, depending on language idiosyncrasies and cultural setting. Measurement invariance refers to the preferred situation where individuals from different subgroups with the same true value of the measured construct have the same probability for each possible response (Mellenbergh, 1989; Millsap & Everson, 1993; Verhagen, 2012; Verhagen et al., 2016) the mathematical function which relates the construct of interest to its indicators (items) must be the same in all compared groups (Borsboom, 2006; Lord, 1980; Mellenbergh, 1989). The situation in which measurement invariance does not hold is referred to as differential functioning. It can be defined at the item level (Differential Item Functioning, DIF), or at the scale level (Differential Test Functioning, DTF; Raju et al., 1995; Stark et al., 2006). DIF is defined as a difference in the probability of choosing a particular answer category for a particular item among individuals having the same level of the construct but belonging to different groups, whereas DTF is defined as a difference in the expected total scores for same-level individuals (Chalmers et al., 2016; Stark et al., 2004, 2006).

When there is DIF or DTF, the sum score has a different meaning for the different subgroups. A sum score like 12 in group A then no longer implies the same thing about the construct as a sum score of 12 in group B. Pooling the sum score data, which is often one of the main objectives of research consortia, then is not meaningful and relevant.

### Measurement Invariance in the Context of Item Response Theory (IRT)

Item response theory (IRT) is a widely used approach for assessing measurement invariance (Meade & Lautenschlager, 2004; Reise et al., 1993; Stark et al., 2006). In the IRT approach, a participants` response on a particular item is modelled as a function of a parameter for the participant`s trait level and parameters for the item (Embretson & Reise, 2000). In psychological research where measurement instruments are commonly based on polytomous items (e.g., Likert-type scale), widely used item parameters are discrimination and threshold item parameters (Embretson & Reise, 2000). The discrimination parameter^2^ refers to the capability of an item to differentiate between persons with similar trait levels and describes the strength of the relationship between trait level and people’s responses to the item (Embretson & Reise, 2000). In the generalized partial credit model (GPCM), where responses are scored 0, 1, …, *C* (where *C* is the highest score category for the item), the threshold parameter is defined as the point on the latent trait continuum where the response probability for two adjacent response categories is equal (Wetzel & Carstensen, 2014). The threshold parameter has no clear equivalence in the factor analysis, but it is most similar to the item intercept, which defines the expected item score in the case when the factor level equals zero (Meade & Lautenschlager, 2004). In contrast to factor analysis, in an IRT model for polytomous items, there are several difficulty parameters (intercepts), more precisely, *C*—1 threshold parameters.^3^ The IRT framework allows us to examine equality of item parameters across groups (Meade & Lautenschlager, 2004; Reise et al., 1993; Stark et al., 2006). In IRT models, an item is invariant, that is, there is no DIF, if its parameters (discrimination and threshold) are equal for all groups (Verhagen, 2012).

In addition, it is important to investigate to which degree the existing differences in item parameters impact the total score on the test (Hambleton, 2006; Pae & Park, 2006; Stark et al., 2006; Suh, 2016). If the test consists of many items and only a few of them have DIF, or DIF effects are weak, then the impact of DIF on DTF may be practically irrelevant (Chalmers et al., 2016). In some cases, even if strong DIF is present, it does not always make an impact on DTF, since a large DIF effect in one direction can exist for one group of items, which can compensate the DIF in another direction due to another group of items (Chalmers et al., 2016). Both DIF and DTF should therefore be investigated. Many studies overlook DTF, which is a pity since data analytical decisions and scientific conclusions are usually based on total test scores rather than individual items (Pae & Park, 2006; Stark et al., 2004).

### Measurement Invariance of CBCL and SDQ

One systematic review that focused on papers that investigated measurement invariance of 26 children and adolescence psychopathology scales (e.g., SDQ and CBCL) showed that children and adolescence psychopathology scales in general lack strong evidence for cross-cultural validity and suitability for cross-cultural comparison (Stevanović et al., 2017). A more detailed analysis reveals that CBCL and SDQ are relatively invariant as long as the differences between the cultures/languages are not large (scales function in very similar or the same way in similar cultures/languages). Research studies focused on cross-country comparison between countries that are distant when it comes to language and culture mostly report the differential functioning (measurement variance) (e.g., Ortuno-Sierra et al., 2015; Stevanović et al., 2015), while in the case of comparing different religion and/or ethnic subgroups within the same country (so they have different religion and/or ethnicity but share the same language and exposed to the same culture), the results shows the presence of measurement invariance (e.g., Guttmannova et al., 2008; Toseeb et al., 2022; Yarnell et al., 2013).

Note that these studies looked into measurement invariance for only one scale at the time, whereas here we are interested in the measurement invariance of a CBCL-SDQ harmonized scale.

### The Aim of the Study and the Research Questions

A previous study demonstrated that the SDQ and CBCL subscales for anxiety/depression and ADHD could be harmonized within an Australian cohort of children (Jović et al., 2022). The aim of this study is to enable researchers to use harmonized measures for the SDQ and CBCL subscales for anxiety/depression and ADHD in the same way across three different cohorts (Australia, the United Kingdom and the Netherlands).

The question of this study is whether the harmonized measures are invariant, that is, whether they function in the same way across three cohorts that differ regarding country, language and age. We will examine whether there are any differences in the CBCL and SDQ item parameters related to anxiety/depression and ADHD across cohorts and to which degree these differences (if they exist) impact the total score on the test, that is, whether they lead to differential test functioning across cohorts.

In order to investigate the above-mentioned, we start from a Bayesian method for modeling measurement non-invariance (Verhagen & Fox, 2013a) that we extend to use on polytomous items and in a relatively small number of groups (cohorts). The extension of this method for using on polytomous items was already used earlier (Van Den Berg et al., 2014), but not explained in detail. We will use estimated item parameters to quantify and visualize the measurement invariance of harmonized measures for anxiety, depression, and ADHD across cohorts at the item level (DIF), but also at the scale level (DTF). Evaluating DTF will be done following a method proposed by Stark et al. (2004).

We will use the data from three cohorts: the Western Australian Pregnancy Cohort-Raine study (Raine; Australia), the Amsterdam Born Children and their Development study (ABCD; The Netherlands), and the Twins Early Development Study (TEDS; The United Kingdom). In all three cohorts, participants filled in the SDQ questionnaire, while the CBCL is filled in by participants from two cohorts (Raine and ABCD).

## Method

### Sample

For the analysis we only used data from children with complete item data on the anxiety/depression and ADHD scales. We used the parent reports on 6930 children (53% girls) from three cohorts on the ADHD scale (Raine = 1551: 738 girls; ABCD = 878: 436 girls; TEDS = 4501; 2489 girls). For anxiety/depression, we used the parent reports on 6877 children (3643 girls) from three cohorts (Raine = 1507 (48% girls), Mean age = 10.60, SD = 0.21; ABCD = 866 (50% girls), Mean age = 11.81, SD = 0.38; TEDS = 4504: (55% girls), Mean = 16.32, SD = 0.68. In the case of twins, one twin was randomly selected. Below we describe the cohorts separately.

### Cohorts

#### The Raine Study

The Raine Study is a longitudinal pregnancy cohort study begun in 1989 and consists of 2900 randomly assigned pregnant women (Chivers et al., 2010; Howard et al., 2011; McKnight et al., 2012; Middeldorp et al., 2019; Newnham et al., 1993). It aimed to study the role that early life events have on later health and to investigate the hypothesis that complications of pregnancy might be prevented by frequent ultrasound scans (McKnight et al., 2012). Those women completed questionnaires at 18 and 34 weeks gestation, as well as at birth, and 1, 2, 3, 5, 8, 10, 14, 17, 18, and 20 years after birth (Howard et al., 2011; McKnight et al., 2012).

For this study, we used the subset which consists of both the CBCL and SDQ parent-filled questionnaires of 2861 children (1417 girls) aged between 10 and 11.5 years (’Generation 2’; Mean = 10.60, SD = 0.21). We excluded from the analysis all participants with missing values on at least one item. The broader Raine Study has ethics approval from The University of Western Australia Human Research Ethics Committee.

#### The Amsterdam Born Children and their Development Study (ABCD)

The Amsterdam Born Children and their Development study (ABCD) is a population-based prospective cohort study that was established in January 2003 and consists of 8266 pregnant women living in Amsterdam (van Eijsden et al., 2011; 12373 pregnant women were invited to participate, but 8266 were included in the study). This study aims to collect data regarding health at birth and in later life as well as data regarding maternal lifestyle and various conditions (medical, psychosocial, environmental) (van Eijsden et al., 2011). The data collection started during pregnancy and is ongoing.

In this study, we used the subset consisting of CBCL and SDQ parent-filled questionnaires of 943 children (472 girls, 471 boys) aged between 11 and 13.5 years (Mean = 11.81, SD = 0.38). In the analysis, we kept only participants who filled in all items (participants with complete data). It is worth mentioning that SDQ and CBCL were filled in during different occasions (SDQ as part of a longer questionnaire, CBCL during a health check). Accordingly, it could be the case that two questionnaires were not filled in by the same parent.

The ABCD Study obtained ethics approval from the Central Committee on Research Involving Human Subjects in the Netherlands.

#### The Twins Early Development Study (TEDS)

The Twins Early Development Study (TEDS) is a population-based twin birth cohort that consists of twins born in the United Kingdom between 1994 and 1996 (Haworth et al., 2013; Rimfeld et al., 2019). The TEDS aimed to obtain a large representative sample in order to investigate genetic and environmental influences on cognitive and behavioral development (Haworth et al., 2013). The collection of the data started when the twins (16810 twin pairs originally included) were about 18 months of age, and followed by collection at 2, 3, 4, 7, 8, 9, 10, 12, 14, and 16 years after birth (Haworth et al., 2013). The total sample consists of 11690 individuals aged between 14.5 and 19 years. We used data from two collection time points when participants were approximately 14 and 16 years old (Mean = 16.32, SD = 0.68). From the total sample, 656 individual twins were excluded (i.e., due to medical reasons or because they were considered as perinatal outliers), leading to a total of 11034 individuals (5517 twin pairs). In order to avoid dependency in the data (twins being from the same family), we randomly selected one twin from each family, which led to a total of 4501 children (2489 girls, 2012 boys) in the case of ADHD, and 4504 children (2491 girls and 2013 boys) in the case of anxiety/depression. The TEDS Study has ethics approval from the University of King`s College London.

### Instruments

#### The Child Behaviour Checklist (CBCL)

We used the 1991 ASEBA (Achenbach System of Empirically Based Assessment) version for the CBCL (age 6–18) by Achenbach (1991). The CBCL operationalizes internalizing and externalizing problems in childhood behavior and it consists of 113 items arranged in eight subscales/dimensions (withdrawn/depression, somatic complaints, anxiety/depression, social problems, thought problems, attention problems, delinquent behavior, and aggressive behavior) (Achenbach et al., 1991; Achenbach et al., 2000). The CBCL is rated on a 3-point Likert type scale with the following answer categories 0—not true, 1—somewhat or sometimes true, and 2—very true or often true. Anxiety/depression is operationalized through 31 items from internalizing problems scale. This scale consists of anxiety/depression, withdrawn/depression, and somatic complaints subscales. ADHD was operationalized through the attention problems subscale consisting of 11 items related to hyperactivity and attention problems. In this study, we used the parent report form of the CBCL questionnaire.

#### The Strengths and Difficulties Questionnaire (SDQ)

We used the 1997 SDQ version by Goodman (1997). The SDQ is a brief behavioral screening questionnaire and it consists of 25 items equally divided across five scales/dimensions (Emotional, Conduct, Hyperactivity/inattention, Peer, and Prosocial problems) (Goodman, 1997, 2001). The SDQ is rated on a 3-point Likert type scale with the following answer categories 0—not true, 1—somewhat true, and 2—certainly true.

Anxiety/depression is operationalized through 5 items from the scale of emotional problems. When it comes to ADHD type of problems, the hyperactivity scale consists of 5 items related to inattention, hyperactivity, and impulsivity, the main symptom domains of ADHD according to DSM-IV classification (American Psychiatric Association, 1994; Goodman & Scott, 1999). We used the parent report form of the SDQ in this study.

### Data analysis

#### Estimation of Item Parameters in Different Cohorts

In order to examine measurement invariance of the CBCL and SDQ items related to anxiety /depression, and ADHD, we use the Bayesian method for modeling measurement non-invariance as proposed by Verhagen and Fox (2013a), and applied earlier by Van den Berg et al. (2014). This method consists of a Bayesian IRT approach for testing differences in item parameters across groups and identifying true differences in means and variances of the latent trait across groups while modelling measurement non-invariance by random item parameters. The IRT approach allows us to include both person (latent trait) and item (discrimination and thresholds) parameters in the analysis, while the Bayesian method enables straightforward estimation of complicated models through hierarchical modeling (Van Den Berg et al., 2014; J. Verhagen, 2012). Unlike other procedures (e.g., likelihood ratio test; Thissen et al., 1993), the Bayesian method for modeling measurement non-invariance does not require an indication of some items as invariant beforehand (Verhagen, 2012).

Verhagen and Fox (2013a) applied this approach to dichotomous data, while Van den Berg et al. (2014) adapted it for use on polytomous items. In both cases, data consisted of a large number of groups (23 in Verhagen & Fox, 2013a; 9 in Van den Berg et al., 2014). Because of the large number of groups, they did not test differences between each pair of groups, but instead assessed the variance of item parameters across cohorts, assuming a hierarchical structure for both the person (scores on the measured construct) and item (discrimination and threshold) parameters (Fox et al., 2007; Fox & Glas, 2001; Verhagen & Fox, 2013a, 2013b; Verhagen, 2012).

In the case of a small number of groups (as we have here), it is generally advised to use fixed effects to model group differences (van den Berg, 2018). We therefore changed the random effects for item parameters into fixed effects by increasing the variance of their prior distributions.

In this study, we used the generalized partial credit model (GPCM) to model item parameters. In the GPCM, the probability of a certain response *c* (*c* = 1, …, *C*) for person *i* (*i* = 1, …, *N*) in group *j* (*j* = 1, …, *J*) on item *k* (*k* = 1, …, *K*) is defined as a function of respondent`s latent trait, ${\theta }_{i}$, item discrimination parameter, ${\widetilde{\alpha }}_{kj}$, and item thresholds for that category, ${\widetilde{\beta }}_{ckj}$, and the ones below it.

$$\mathcal{P}({Y}_{ijk}=c\mid {\eta }_{ijck})=\text{exp}(\sum_{c\in C}({\eta }_{ijck}))$$

$${\eta }_{ijck}={\widetilde{\alpha }}_{kj}({\theta }_{ij}-{\widetilde{\beta }}_{ckj})$$

The item and person parameters are estimated through a Markov Chain Monte Carlo (MCMC) procedure (Van Den Berg et al., 2014; Verhagen & Fox, 2013a, 2013b). The person parameters (latent trait values) are modeled to be normally distributed around their group mean, ${\mu }_{{\theta }_{j}}$, with precision ${\tau }_{j}$ (precision ${\tau }_{j}$ is inverted variance).

$${\theta }_{i}\sim \mathcal{N}({\mu }_{{\theta }_{j}},{\tau }_{j})$$

In the case of fixed effects for the group-specific parameters, appropriate prior distributions for the group means are normal priors with very large variance parameters (low precision). Accordingly, the group means in our model were normally distributed and centered around 0 with the precision of 0.1 (variance of 10) in order to get quasi-fixed effects for groups.

$${\mu }_{{\theta }_{j}}\sim \mathcal{N}(0,.1)$$

The precision ${\tau }_{j}$ of the person parameters ${\theta }_{i}$ is given a gamma prior distribution with a shape parameter of 1 and a scale parameter of 0.1. This precision is the inverse of the within-group variance ${\sigma }_{j}$.

$${\tau }_{j}\sim \Gamma (1,.1)$$

The multilevel structure on the item parameters consists of group-specific item parameters ${\widetilde{\xi }}_{kj}$, and each set of item parameters per item per group comes from a multivariate normal distribution with specific item mean and specific item covariance (precision). In this study, we have one discrimination parameter and two threshold parameters per item. Accordingly, the group-specific item parameters ${\widetilde{\xi }}_{kj}$ are consisting of group-specific discrimination parameter ${\widetilde{\alpha }}_{kj}$ and two group-specific threshold parameters (${\widetilde{\beta }}_{1kj}$ and ${\widetilde{\beta }}_{2kj}$) for each item in a certain group. The group-specific item parameters are multivariate normally distributed around general item parameters ${\xi }_{k}$, with uninformative prior $\mathcal{Q}$ for a precision matrix.

$${\widetilde{\xi }}_{kj}\sim \mathcal{N}({\xi }_{0},\mathcal{Q})$$

The general item parameters ${\xi }_{k}$ are assumed to be multivariate normally distributed around mean ${\xi }_{0}$, with covariance matrix $\Sigma {\xi }_{k}$.

$${\xi }_{k}\sim \mathcal{N}({\xi }_{0},\Sigma {\xi }_{k})$$

The ${\xi }_{0}$ is assumed to be multivariate normally distributed around the overall parameter means ${\mu }_{k}$, with variance described by precision matrix $\mathcal{R}$ for the discrimination and each threshold parameter. An inverse Wishart prior distribution is chosen for the covariance matrix $\Sigma {\xi }_{k}$.

$${\xi }_{0}\sim \mathcal{N}({\mu }_{k},\mathcal{R})$$

$${\Sigma }_{{\xi }_{k}}\sim \mathcal{I}\mathcal{W}(\mathcal{R},d)$$

$$d=\mathcal{C}$$

$${\mu }_{k\beta }=0$$

$${\mu }_{k\alpha }=1$$

The Inverse Wishart distribution is specified with a $\mathcal{C}x\mathcal{C}$ scale matrix $\mathcal{R}$, where $\mathcal{C}$ is the number of item parameters, and with a number of degrees of freedom d. The degrees of freedom must be higher than $\mathcal{C}-1$ (Schuurman et al., 2016). It is assumed that the overall parameter means are equal to 0 in the case of threshold parameters, and 1 in the case of the discrimination parameter.

The model is identified by a restriction in such a way that the overall mean threshold in each group is equal to 0 and the product of the discrimination parameters is equal to 1 within each group.

One cohort can have higher scores on the test because of a higher group-mean latent trait or because all items have lower threshold parameters for this cohort (Verhagen, 2012). The restriction that the threshold parameters are 0 on average, allows us to identify the means of the thetas. Similarly, more variance of one cohort on the test can be a consequence of a larger variance on the latent distribution or smaller discrimination parameters for this cohort. Accordingly, the variance of the latent scale can be identified by restricting the product of the discrimination parameters to 1 (van den Berg et al., 2014; Verhagen, 2012).

In the Supplementary Material, we include examples and explain the logic of the model in more detail.

#### Quantification and Visualization of Measurement Invariance at the Scale Level

We will quantify the DTF using the method proposed by Stark et al. (2004). This method is an improved version of one of the most prominent methods for calculating the DTF proposed by Raju et al. (1995). It enables researchers to quantify the DTF and to express its degree in raw test scores. Stark et al. (2004) proposed an estimation of the expected total test scores for participants from one cohort based on different sets of item parameters. In other words, in the first case, the expected total test score for the participant from the reference group will be estimated based on item parameters from the reference group, while in the second case it will be estimated based on item parameters from the focal group. We will have two expected total test scores for each participant—one estimated using item parameters from the reference group and another estimated using item parameters from the focal group. Accordingly, we will have two Test Characteristic Curves (TCC)—one based on item parameters from the reference group and another based on item parameters from the focal group.

For expressing the amount of DTF, Stark et al. (2004) used the DTFR parameter. The DTFR measure is similar to the DTF parameter proposed by Raju et al. (1995) and is the expected difference between the TCCs:

$$DTFR=E(TC{C}_{R}-TC{C}_{F})$$

$TC{C}_{R}$ is based on the item parameters from the reference group, while $TC{C}_{F}$ is based on item parameters from the focal group.

The greater the absolute value of DTFR, the greater the differential functioning of a test. While an absolute value of DTFR tells us what amount of DTFR is present, the sign of the DTFR tells us which group has higher expected test scores due to differential test functioning. Stark et al. (2004) proposed to subtract the values of expected test scores of the focal group from values of expected test scores of the reference group ($TC{C}_{R}-TC{C}_{F}$). Consequently, a positive value of DTFR means that the expected test scores based on item parameters from the focal group are lower than expected test scores based on item parameters from the reference group, while a negative value means that the scores based on item parameters from the focal group are higher than scores based on item parameters from the reference group. Since this is a bit counter-intuitive, $TC{C}_{R}-TC{C}_{F}$ we will use $TC{C}_{F}-TC{C}_{R}$ (Raju et al., 1995).

$$DTFR=E(TC{C}_{F}-TC{C}_{R})$$

Based on DTFR, we can obtain an effect size by using (Stark et al., 2004):

$${d}_{DTF}=\frac{DTFR}{S{D}_{F}}$$

Stark et al. (2004) also proposed another interesting parameter – IMPACT. DTFR is the difference between groups caused by differential test functioning, while IMPACT represents the true mean difference between groups, that is, a component of the observed mean difference that is not caused by differential test functioning. Both of them are forming an Observed Mean Difference (OMD).

$$OM{D}_{(F-R)}={M}_{(F)}-{M}_{(R)}$$

$$OM{D}_{(F-R)}=DTFR+IMPACT$$

$$IMPACT=OM{D}_{(F-R)}-DTFR$$

We will calculate an IMPACT score to determine the amount of difference between sum scores that is caused by the true mean difference between groups. Because of the specific restriction that the threshold parameters are on average 0 in the Bayesian modelling approach, the DTFR should be close to 0, once the groups are allowed to have different trait means (the IMPACT component of the OMD).

We will visualize DTF in order to gain more precise insights about its presence for different values of the latent trait. We will use the method proposed by Stark et al. (2004). Their method is based on using the Test Characteristic Curve (TCC). The test characteristic curve is the functional relation between the true score and the latent trait scale (Baker & Kim, 2017). Stark et al. (2004) calculated expected sum scores for any point on the latent trait scale from −3 to 3 in order to plot them, that is, to plot TCC, where the scale is defined by a variance of 1. By visualizing the TCC, the researchers are able to find the corresponding test score for any level of the latent trait. Since our scale for person parameters is defined by the restriction that the product of the discrimination parameters is equal to 1, we will instead visualize TCCs using item parameters from different cohorts to visualize expected sum scores for any point on the latent trait scale in the range between minimum and maximum observed theta in the sample.

## Results

We analyzed anxiety/depression and ADHD scales separately. In the first part of the analysis, we used the Bayesian method for modeling measurement non-invariance, estimated item parameters across cohorts and identified true differences in means and variances of anxiety/depression and ADHD across cohorts. Next, we used estimated item parameters to quantify and visualize the measurement invariance at the scale level (DTF).

### ADHD

**Fig. 1:**
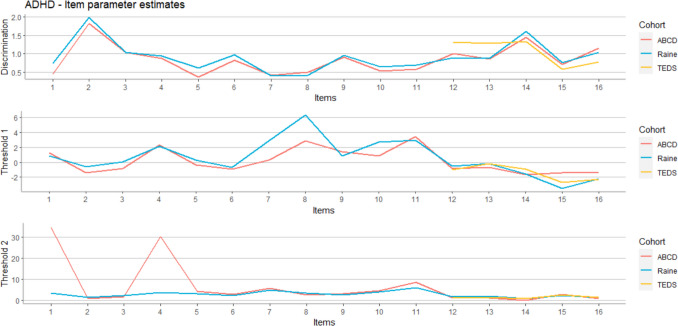
Estimated item parameters (discrimination and threshold) for 16 ADHD items (1–11 CBCL, 12–16 SDQ) for three different cohorts

We estimated parameters based on participants’ responses on 11 CBCL and 5 SDQ items for Raine and ABCD cohorts, while for the TEDS cohort we used only 5 SDQ items because in this cohort we did not have participants’ responses on CBCL items. After a burn-in phase of 1000 iterations, the characterization of the posterior distribution was based on a total of 10000 iterations. The values of discrimination and threshold parameters in three different cohorts are presented graphically in Fig. 1 and numerically in the Supplementary materials (Table S1). The discrimination parameters were largely the same in both Raine and ABCD cohorts for all CBCL items (first 11 items), while in the case of threshold parameters we observed a few striking differences (Fig. 1 and Table S1). The threshold 1 parameter for the two items related to nervousness (item 7—“CBCL nervous or tense” and item 8—“CBCL nervous movements”) was slightly higher in the Raine than in the ABCD cohort.

**Fig. 2:**
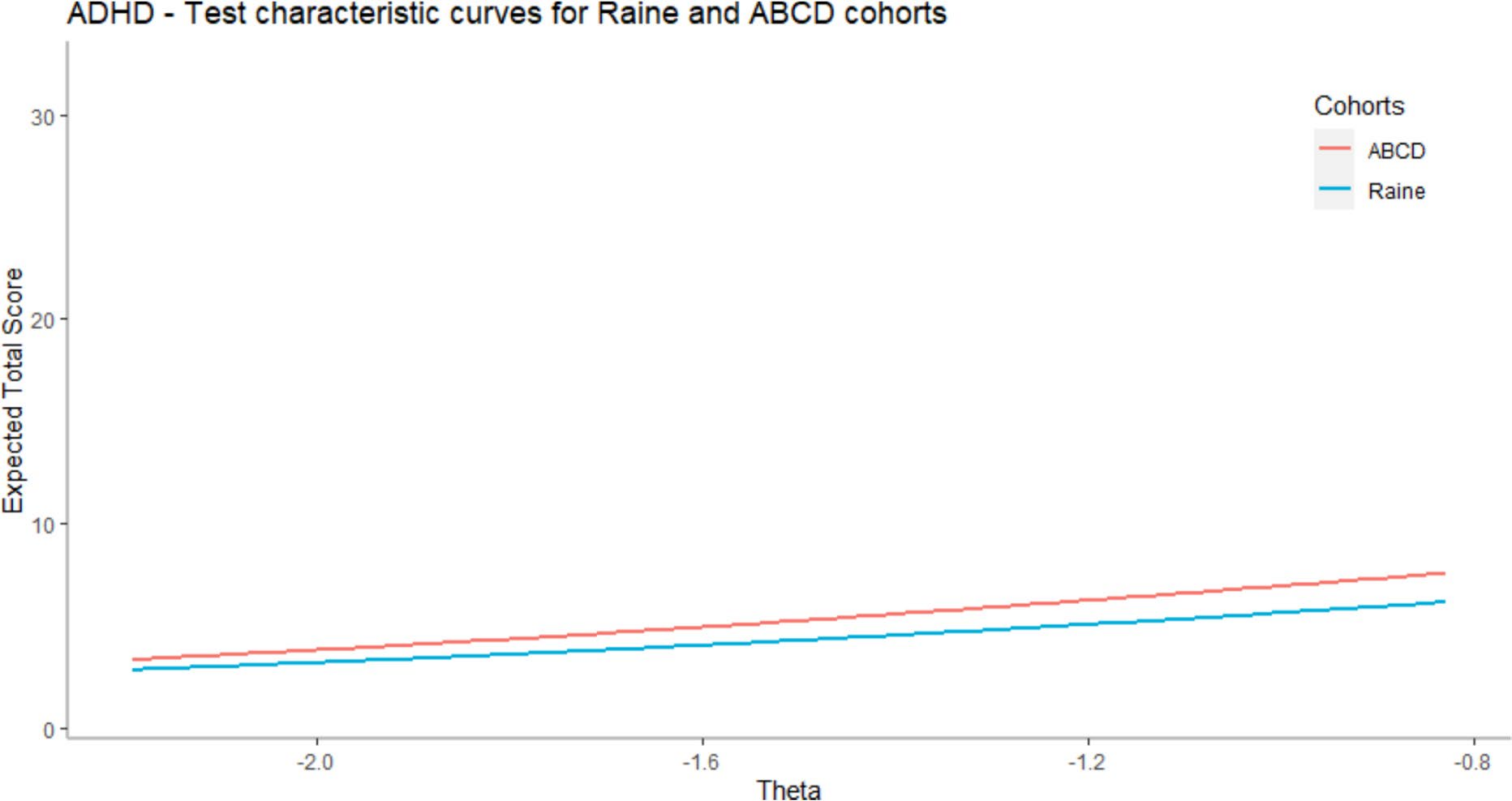
Comparison of TCCs for Raine and ABCD cohorts (all items). For context, the expected total scores in the sample were between 2.82 and 7.54. The maximum theoretically possible score was 32

In the case of the threshold 2 parameter, the situation was the opposite. Two items (item 1 – “CBCL acts too young” and item 4 – “CBCL confused”) were much higher in the ABCD than in the Raine cohort. The threshold parameters for the SDQ items (items from 12 to 16), were almost the same for all three cohorts. We observed that discrimination parameters are slightly higher in the TEDS cohort than in the Raine and ABCD cohorts for the two items (item 12—“SDQ restless” and item 13—“SDQ constantly fidgeting”) Fig. 2.

By using Bayesian modeling we considered these deviations in item parameters across cohorts and estimated means per cohort while controlling for any measurement non-invariance (Table 1). The highest mean is observed for the TEDS cohort, then for the Raine cohort, while the lowest mean is observed in the case of the ABCD cohort.

**Table 1:** Estimated means and their standard errors (i.e., posterior standard deviation) of the ADHD latent trait after taking into account measurement non-invariance across cohorts

Cohort	Minimum	Maximum	Mean	Standard error
ABCD	−2.19	−1.54	−1.86	.08
Raine	−1.68	−1.24	−1.46	.06
TEDS	−1.03	-.83	-.92	.03

In the second part of the analysis, we examined the practical significance of the detected differences in item parameters between groups. We quantified, and visualized the DTF in order to examine if, and to which degree, the observed differences in item parameters impact the measurement invariance on the test level. First, we compared different cohorts and presented the mean test scores, standard deviations, and DTFR in Table 2. It is worth mentioning that we were able to use the whole scale only in the case of comparison between Raine and ABCD cohorts, but not in the case when we compared these cohorts with the TEDS cohort because, in the TEDS cohort, we have only participants’ responses on 5 SDQ items.

**Table 2:** ADHD – DTF between cohorts

**Cohorts (F-R)**	**TS**	*M*_*F*_	*SD*_*F*_	*M*_*R*_	*SD*_*R*_	**OMD**	**DTFR**	**d***DTF*	**IMPACT**
Raine-ABCD	32	21.7	5.02	21.6	4.86	0.1	−0.55	−0.11	0.65
Raine-TEDS	10	8.13	2.35	8.56	2.31	−0.43	0.22	0.09	−0.65
ABCD-TEDS	10	7.54	2.45	8.56	2.31	−1.02	−0.12	−0.05	−0.9

F = Focal group, R = Reference group, TS = Maximum possible test score, OMD = Observed Mean Difference

**Fig. 3:**
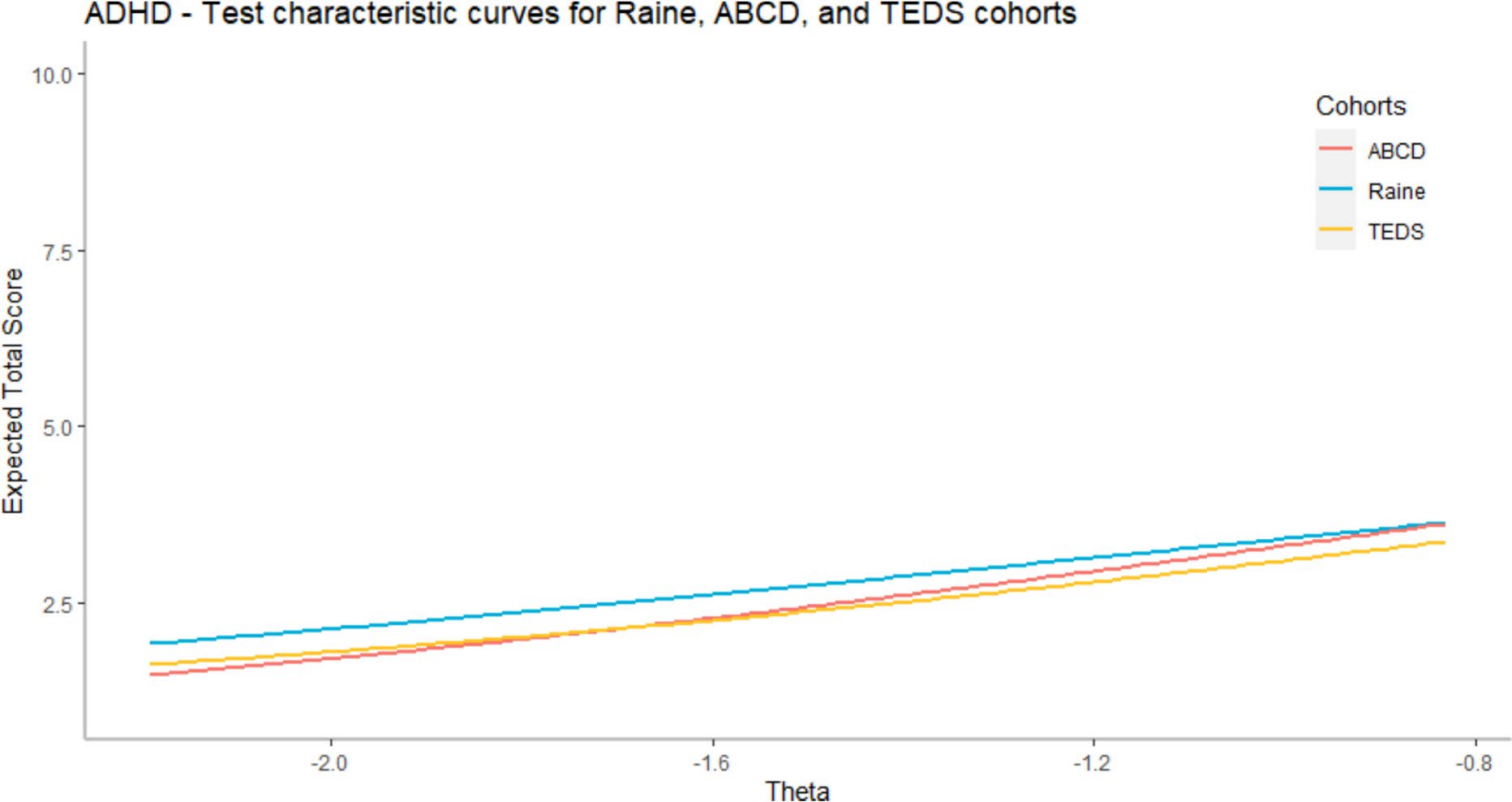
Comparison of TCCs for Raine, ABCD and TEDS cohorts (SDQ items). For context, the expected total scores in the sample were between 1.47 and 3.63. The maximum theoretically possible score was 10

**Fig. 4:**
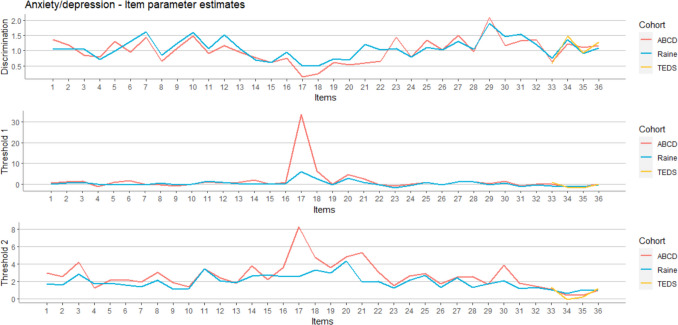
Estimated item parameters (discrimination and threshold) for 36 anxiety/depression items (1–31 CBCL, 32–36 SDQ) for three different cohorts

In the first case, Raine and ABCD cohorts are compared by using all ADHD items (16 items), while in the case of the other two comparisons (Raine and TEDS, and ABCD and TEDS), only ADHD items from the SDQ scale are used (5 items) because in the TEDS cohort we do not have participants` responses on CBCL scale. The results showed that the DTF is negligible in all three comparisons. In the first case, we compared the Raine and ABCD cohorts. The maximum score on the test is 32 and the score of participants from the Raine group is, on average, 0.55 underestimated in comparison with participants from the ABCD cohort (effect size = −0.11). In the situation when we compared Raine and TEDS cohorts, the results showed that a similar amount of negligible DTF is present. The scores of participants from the Raine cohort are overestimated for 0.22 (effect size = 0.09). In the third case, the scores of participants from ABCD cohorts are slightly (0.12) underestimated in comparison with participants from the TEDS cohort (effect size = −0.05). When it comes to the IMPACT, the results showed that participants from the Raine cohort have, on average, 0.65 higher test scores caused only by true mean differences in the latent trait). On the other side, their test scores are, on average, 0.65 lower than the scores of participants from the TEDS cohort. In the third comparison (ABCD and TEDS), true mean differences in the latent trait caused lower scores on the test among participants from the ABCD group. Their scores are, on average, 0.9 lower than scores of participants from the TEDS cohort due to differences in the level of the latent trait. After that, we visualized TCCs in order to show what amount of DTF is present for any point on the latent trait scale in the range between minimum and maximum observed theta in the sample. First, we compared the expected test scores of participants from the Raine and ABCD cohort using all items. After that, we compared all three cohorts using only SDQ items, because for the TEDS cohort we only have participants’ responses on SDQ items (5 items). In both figures (Figs. 3 and 4), the results showed that differences in expected test scores due to DTF are negligible. Note that on the y-axis, we presented the range between the minimal and maximal theoretically possible scores on the test in order to show differences in test scores in the context of the whole range of possible scores on the test. Another possible solution is to present the range between the minimal and maximal observed scores on the test. But, in that case, even small differences in test scores may seem big on the graphical representation. We decided to use the solution with minimal and maximal possible scores because in that case the graphical presentation is much more informative and it is a valuable addition to the numerically presented DTF.

### Anxiety/Depression

We estimated parameters based on participants’ responses on 31 CBCL and 5 SDQ items for Raine and ABCD cohorts, while for the TEDS cohort we used only 4 SDQ items because in this cohort we did not have participants` responses on CBCL items as well as responses on one SDQ item (“SDQ often unhappy/tearful”). After a burn-in phase of 1000 iterations, the characterization of the posterior distribution was based on a total of 10000 iterations. The values of discrimination and threshold parameters in three different cohorts are presented graphically and numerically in Fig. 4 and Table S2. The discrimination parameters were largely the same in both Raine and ABCD cohorts for all CBCL items (first 31 items), while in the case of threshold parameters we observed striking differences for only one item. Both threshold parameters for item “CBCL no cause eye problems” (item 17) were higher in the ABCD than in the Raine cohort. Both discrimination and threshold parameters for the SDQ items (items from 32 to 36), were almost the same for all three cohorts.

We considered these deviations in item parameters across cohorts and estimated means and variances per cohort while controlling for any measurement non-invariance (Table 3). The highest mean is observed for the TEDS cohort, while the means of Raine and ABCD cohorts are the same.

**Table 3:** Estimated means and standard errors of the anxiety/depression latent trait after taking into account measurement non-invariance across cohorts

Cohort	Minimum	Maximum	Mean	Standard error
ABCD	−2.24	−1.70	−1.95	.08
Raine	−2.19	−1.66	−1.95	.06
TEDS	−1.51	−1.25	−1.37	.03

In the second part of the analysis, we investigated, quantified, and visualized the DTF in order to examine if, and to which degree, the observed DIF impacts the measurement invariance on the test level, that is, DTF. First, we compared different cohorts and presented the mean test scores and their standard deviations for different groups, mean difference between them, as well as DTFR, and an IMPACT in Table 4. As in the case of ADHD, we were able to use the whole scale only in the case of comparison between Raine and ABCD cohorts, but not in the case when we compared these cohorts with the TEDS cohort because, in this cohort, we have only participants` responses on 4 SDQ items.

**Table 4:** Anxiety/depression – DTF between cohorts

**Cohorts (F-R)**	**TS**	*M*_*F*_	*SD*_*F*_	*M*_*R*_	*SD*_*R*_	**OMD**	**DTFR**	**d***DTF*	**IMPACT**
Raine-ABCD	72	43.4	7.09	42.9	6.51	0.5	0.28	0.04	0.22
Raine-TEDS	8	5.53	1.62	6.26	1.90	−0.73	−0.04	−0.02	−0.69
ABCD-TEDS	8	5.53	1.69	6.26	1.90	−0.73	−0.08	−0.05	−0.65

F = Focal group, R = Reference group, TS = Maximum possible test score, OMD = Observed Mean Difference

In the first case, Raine and ABCD cohorts are compared by using all anxiety/depression items (36), while in the case of the other two comparisons (Raine and TEDS, and ABCD and TEDS), only 4 items from the SDQ scale are used because in the TEDS cohort we do not have participants’ responses on CBCL scale and one item from the SDQ scale. The results showed that in the case of anxiety/depression the DTF is even smaller and more negligible than for ADHD. The DTF between Raine and ABCD cohorts is 0.28 (effect size = 0.04), while in the case of two other comparisons it is even smaller −0.04 (effect size = −0.02) between Raine and TEDS and −0.08 (effect size = −0.05) between ABCD and TEDS. An IMPACT showed that participants from the Raine cohort have 0.22 higher test scores than participants from the ABCD caused by the difference in the latent trait and 0.69 lower scores from participants from the TEDS cohort. Participants from the TEDS cohort also have slightly higher test scores (0.65 higher) than participants from the ABCD cohort.

**Fig. 5:**
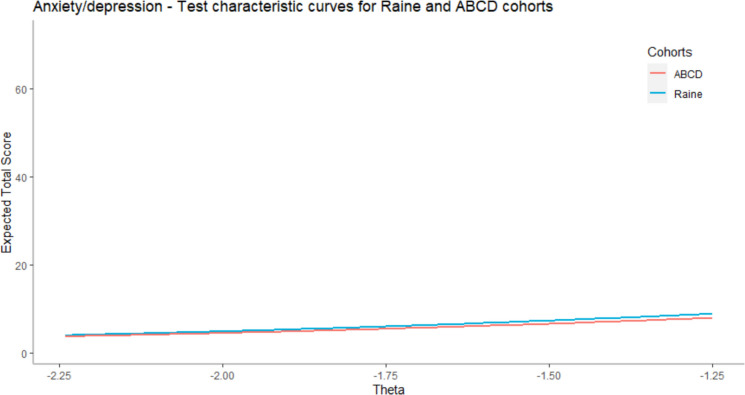
Comparison of TCCs for Raine and ABCD cohorts (all items). For context, the expected total scores in the sample were between 3.74 and 8.93. The maximum theoretically possible score was 72

**Fig. 6:**
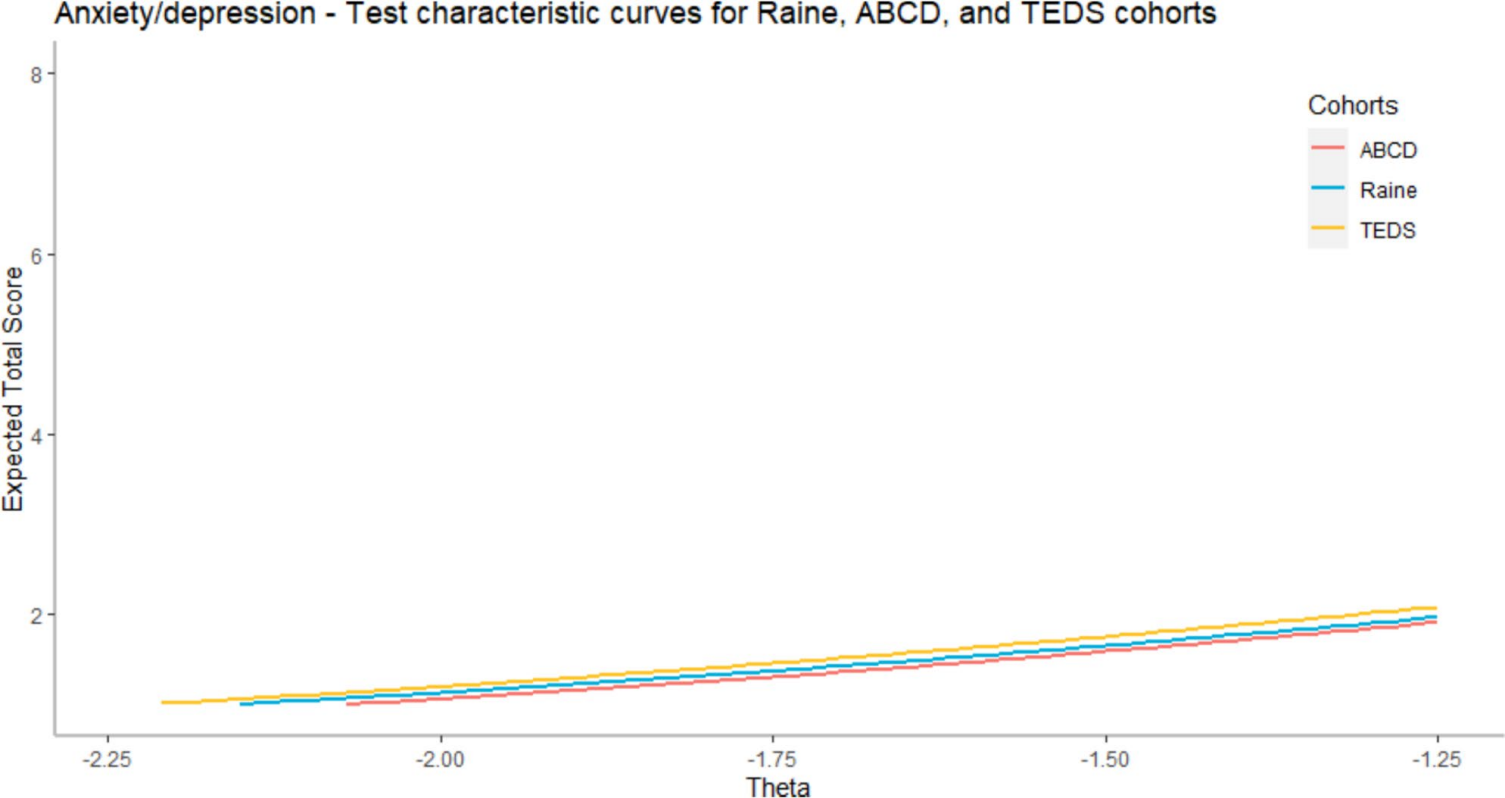
Comparison of TCCs for Raine, ABCD and TEDS cohorts (SDQ items). For context, the expected total scores in the sample were between 0.86 and 2.08. The maximum theoretically possible score was 10

After that, we visualized the TCCs of compared groups. In Fig. 5, we compared the expected test scores of participants from the Raine and ABCD cohort using all items (31 CBCL and 5 SDQ). In Fig. 6, we compared all three cohorts using 4 SDQ items, because for the TEDS cohort we have only participants’ responses on those 4 SDQ items. In both cases, the results showed that differences in expected test scores caused by DTF are negligible. Note that on the y-axis, we presented the range between the minimal and maximal theoretically possible scores on the test.

## Discussion

This study aimed to examine whether the harmonized measures for anxiety/depression and ADHD in 10-year-old Australian children (Jović et al., 2022) were measurement invariant across countries, languages, and different age groups. In this study, we investigated if there is measurement invariance between harmonized scores from Australia, the Netherlands and the United Kingdom. Results showed that the model used for harmonizing SDQ and CBCL measures in Australian 10-year-old children (Jović et al., 2022) can also be used in cohorts from other countries and older ages. The use of harmonized scores enables researchers to increase the sample size, generalizability, validity, or statistical power of research results (Fortier et al., 2010; Fortier et al., 2011; Hamilton et al., 2011; Jović et al., 2022; Smith-Warner et al., 2006; Thompson, 2009; Van Den Berg et al., 2014). Accordingly, we advise using harmonized scores whenever possible, at least for the age groups and countries studied here.

This conclusion is based on the use of an extension of the Bayesian method for modeling measurement non-invariance proposed by Verhagen and Fox (Verhagen, 2012; Verhagen & Fox, 2013a) that we extended for polytomous items and where we used a fixed effects approach given the small number of groups (Van Den Berg et al., 2014; Verhagen & Fox, 2013a).

The results showed that item parameters are largely invariant across all three cohorts, both for the anxiety/depression and the ADHD items. Consequently, the results showed that the method and model used for harmonization of SDQ and CBCL on the Australian sample can also be used in cohorts from the Netherlands and the United Kingdom. Only in very few cases there were some striking differences in item parameters: two CBCL ADHD items have higher threshold 2 parameters in the ABCD than in the Raine cohort (“acts too young”, “acts confused”).

Next, we used the method proposed by Stark et al. (2004) to examine if, and to which degree, the observed differences in item parameters lead to DTF. The results showed that the observed differences in item parameters do not impact the measurement invariance at the scale level. The visualization of the DTF showed that the differences are negligible both for the anxiety/depression and ADHD scales. This was to be expected, given the restrictions in the Bayesian modelling approach. It demonstrates the attractiveness of the modelling approach, since you end up with parameters for persons and items in such a way that estimated trait values will be free from bias due to DIF. Using the estimated trait values instead of the observed sum scores yields harmonized scores. The only assumption that one needs to accept is that the average thresholds are 0 (i.e. that the average difficulty across all items is the same groups).

The mostly absent DIF is in line with other studies that showed that there is measurement variance across countries, but mostly for countries that are more distant when it comes to language and culture (Ortuno-Sierra et al., 2015; Stevanović et al., 2015; Stevanović et al., 2017). In our case, two out of three cohorts use the same language—English (Raine and TEDS), while two cohorts are from Europe (TEDS and ABCD). Moreover, in all three cohorts, the Western culture is dominant.

We also estimated trait means across cohorts while controlling for any measurement non-invariance. For both anxiety/depression and ADHD, the highest mean is observed in the TEDS cohort. It is worth mentioning that scores in the TEDS cohort were estimated based only on SDQ items, but these true differences in mean scores do not depend on the number of items. The means of Raine and ABCD cohorts are the same in the case of anxiety/depression, while in the case of ADHD the mean of the Raine cohort is slightly higher than the mean of the ABCD cohort. These differences in true means also lead to differences in sum scores between cohorts. The IMPACT, which tells us about the amount of difference in sum scores that is caused by’true’ mean difference between cohorts, showed that, on average, the sum score of the participants from the TEDS cohort is slightly higher than the sum score of participants from the other two cohorts for both anxiety/depression and ADHD. That can be explained by differences in the age structure of the participants in different cohorts (mean age: TEDS = 16.32, Raine = 10.60, ABCD = 11.81), since the research results shows that prevalence rates of both anxiety/depression and ADHD are approximately 60% higher among children between 12 and 17 years than among children younger than 12 years (Centers for Disease Control and Prevention (CDS), 2020, 2021).

### Theoretical Implications

This study explains in detail the extension of the Bayesian method for modeling measurement non-invariance to polytomous items. Furthermore, the literature review showed that there is a lack of research that investigate measurement invariance of harmonized SDQ and CBCL measures, and this study contributes to the literature by showing that the harmonized measures for the SDQ and CBCL subscales for the anxiety/depression and ADHD are invariant across cohorts that differ in the country of origin, language and age (Australia, the Netherlands, the United Kingdom).

### Practical Implications

The model used for harmonizing SDQ and CBCL measures in Australian 10-year-old children (Jović et al., 2022) can also be used in cohorts from the Netherlands and the United Kingdom. This research supports the use of harmonized measures in cross-cultural mental health studies which enables better generalizability and validity of research results. Accordingly, based on the results from this study, we advise using harmonized scores whenever possible, at least for the age groups and countries studied here.

### Limitations

When it comes to the limitations of this study, some of them are related to the samples that we used, while some of them are related to the existing methodology for quantification and visualization of DTF. In this study, we used cohorts that are very similar in terms of language and culture. Besides, the results are based on the data collected on the general population.

In addition, there are several limitations of this study that are related to the method used for quantification and visualization of DTF. The method that we used is proposed by Stark et al. (2004). This method is currently one of the most comprehensive methods for the investigation of DTF. The biggest value of this method is the fact that authors advocate using effect sizes in addition or instead of statistical significance because ‘statistical’ significance of DTF does not imply ‘practical’ significance. Statistical significance refers to whether the results could have arisen by chance (sampling variability), while practical significance is focused on whether the results are consequential in the real world (Kirk, 1996). However, this method has the important limitation that the variation in expected scores is not visualised and it only gives us expected means but not variances of the expected scores.

Another limitation is caused by the neglect of sampling variability of item parameter estimates. Namely, this visualization method does not take into account the uncertainty of the item parameter estimates in different groups. This uncertainty should preferably also be visualized in the DTF plots. This could be done by drawing samples from the posterior distribution, but this was outside the scope of this paper. We generally discovered DIF with only very limited practical implications. We generally conclude that the scales can be harmonized, preferably while taking into account DIF through Bayesian modelling, but even when not, the practical implications are small when this is not done.

In general, there is a lack of methods for quantification and visualization of measurement variance at the scale level, and, as we can see, there is no method that is comprehensive enough. Moreover, visualization of DTF in the method proposed by Stark et al. (2004) is used only for illustrative purposes, while the decisions are still based on various statistical tests.

### Future Research Directions

The cohorts we used in this study are quite similar in terms of language and culture. In future research, it would be useful to investigate if the measurement invariance extends to cohorts that are not part of Western culture.

Moreover, the results in this study are based on the data collected on general population. It could be interesting to investigate if the measurement invariance also exists on the sample of participants from the clinical population.

There is also a lack of a comprehensive method for quantification and visualization of measurement variance at the scale level. It would be useful to develop a new and comprehensive visualization-based method for investigation and quantification of measurement invariance, especially at the scale level, that overcomes disadvantages that are present in the existing methods and that will be easy to understand and interpret.

## Conclusion

The results of the previous study showed that the SDQ and CBCL subscales for anxiety/depression and ADHD can be harmonized on a sample of children from Australia (Jović et al., 2022). There is a lack of research that investigates the measurement invariance for the harmonized SDQ and CBCL subscales for anxiety/depression and ADHD. Accordingly, in this study, we aimed to investigate if harmonized measures for the SDQ and CBCL subscales for anxiety/depression and ADHD can be used in the same way across three different cohorts (Australia, the United Kingdom and the Netherlands). In order to do so, we had to check if harmonized measures are invariant, that is, if they function in the same way across cohorts.

First, we explained in detail how the Bayesian method for modeling measurement non-invariance can be used for polytomous items. Then, we implemented it to the SDQ and CBCL anxiety/depression and ADHD items to investigate if item parameters are invariant across cohorts. The results showed that item parameters are largely invariant across all three cohorts, both for the anxiety/depression and the ADHD items. The DTF statistical analysis as well as the visualization of the DTF showed that even in the cases where there are some differences in the functioning of individual items, the scores on the scale level are not impacted.

Accordingly, the model used for harmonizing CBCL and SDQ measures in Australian 10-year-old children (Jović et al., 2022) can also be used in cohorts from the Netherlands and the United Kingdom.

## Supplementary Information

Below is the link to the electronic supplementary material.

Supplementary file1 (DOCX 87 KB)

## Data Availability

The data analyzed in this study is subject to the following licenses/restrictions: Data is not publicly available because it is owned by Raine Study, TEDS and ABCD and permission for getting and analyzing it should be obtained from them.

## Footnotes

1 Invariant in this context means that the scale functions in the same way across countries, languages, cultures, age groups, etc.

2 The discrimination parameter can be conceptually and functionally compared to a factor loading in factor analysis (Meade & Lautenschlager, 2004; van den Berg et al., 2007).

3 Accordingly, for a 3-point scale, we have two threshold parameters, first between categories 1 and 2, and second between categories 2 and 3.
