# Supplementary material for: Harmonization of SDQ and ASEBA Phenotypes: Measurement Variance Across Cohorts
Source: J Psychopathol Behav Assess. 2025 Mar 7;47(1):27. doi: 10.1007/s10862-025-10204-0 (PMC11889055; doi:10.1007/s10862-025-10204-0)
Supplement: Supplementary file 1 — Supplementary file1 (DOCX 87 KB) [file 10862_2025_10204_MOESM1_ESM.docx]

# Harmonization of SDQ and ASEBA phenotypes: measurement variance across cohorts

# Supplementary material

## Tables

Table S1 Estimated item parameters (discrimination and threshold) for 16 ADHD items for three different cohorts

|  | ABCD | | | Raine | | | TEDS | | |
| --- | --- | --- | --- | --- | --- | --- | --- | --- | --- |
| Item | *α* | *β*_1_ | *β*_2_ | *α* | *β*_1_ | *β*_2_ | *α* | *β*_1_ | *β*_2_ |
| 1 | 0.747 | 0.839 | 3.409 | 0.454 | 1.289 | 34.808 | NA | NA | NA |
| 2 | 1.987 | -0.565 | 1.442 | 1.824 | -1.436 | 0.709 | NA | NA | NA |
| 3 | 0.030 | 0.055 | 2.294 | 1.032 | -0.835 | 1.711 | NA | NA | NA |
| 4 | 0.940 | 2.145 | 3.749 | 0.881 | 2.287 | 30.272 | NA | NA | NA |
| 5 | 0.606 | 0.309 | 3.044 | 0.377 | -0.390 | 4.407 | NA | NA | NA |
| 6 | 0.970 | -0.660 | 2.301 | 0.822 | -0.923 | 2.941 | NA | NA | NA |
| 7 | 0.393 | 2.840 | 4.883 | 0.425 | 0.330 | 5.604 | NA | NA | NA |
| 8 | 0.423 | 6.326 | 3.303 | 0.495 | 2.850 | 2.479 | NA | NA | NA |
| 9 | 0.962 | 0.887 | 2.452 | 0.906 | 1.397 | 3.150 | NA | NA | NA |
| 10 | 0.642 | 2.697 | 3.934 | 0.533 | 0.860 | 4.541 | NA | NA | NA |
| 11 | 0.695 | 2.928 | 5.966 | 0.582 | 3.410 | 8.471 | NA | NA | NA |
| 12 | 0.897 | -0.542 | 1.829 | 0.998 | -0.830 | 1.126 | 1.313 | -1.031 | 0.980 |
| 13 | 0.882 | -0.192 | 1.944 | 0.852 | -0.653 | 1.134 | 1.285 | -0.228 | 1.343 |
| 14 | 1.616 | -1.601 | 0.764 | 1.450 | -1.636 | -0.039 | 1.333 | -0.923 | 0.889 |
| 15 | 0.757 | -3.480 | 2.186 | 0.708 | -1.436 | 2.773 | 0.573 | -2.724 | 2.638 |
| 16 | 1.040 | -2.176 | 1.268 | 1.160 | -1.381 | 0.941 | 0.781 | -2.285 | 1.341 |

Table S2 Estimated item parameters (discrimination and threshold) for 36 anxiety/depression items for three different cohorts

|  | ABCD | | | Raine | | | TEDS | | |
| --- | --- | --- | --- | --- | --- | --- | --- | --- | --- |
| Item | *α* | *β*_1_ | *β*_2_ | *α* | *β*_1_ | *β*_2_ | *α* | *β*_1_ | *β*_2_ |
| 1 | 1.073 | 0.073 | 1.762 | 1.362 | 0.507 | 3.004 | NA | NA | NA |
| 2 | 1.043 | 0.448 | 1.587 | 1.189 | 1.022 | 2.573 | NA | NA | NA |
| 3 | 1.035 | 0.686 | 2.860 | 0.847 | 1.431 | 4.191 | NA | NA | NA |
| 4 | 0.710 | -0.209 | 1.728 | 0.790 | -1.273 | 1.263 | NA | NA | NA |
| 5 | 0.987 | -0.280 | 1.782 | 1.292 | 0.678 | 2.136 | NA | NA | NA |
| 6 | 1.306 | 0.065 | 1.600 | 0.951 | 1.746 | 2.223 | NA | NA | NA |
| 7 | 1.612 | -0.276 | 1.356 | 1.440 | -0.065 | 1.935 | NA | NA | NA |
| 8 | 0.844 | 0.541 | 2.134 | 0.656 | -0.228 | 3.067 | NA | NA | NA |
| 9 | 1.247 | -0.316 | 1.141 | 1.078 | -0.964 | 1.860 | NA | NA | NA |
| 10 | 1.595 | -0.073 | 1.193 | 1.476 | 0.044 | 1.398 | NA | NA | NA |
| 11 | 1.065 | 1.502 | 3.470 | 0.902 | 1.229 | 3.470 | NA | NA | NA |
| 12 | 1.513 | 0.754 | 2.065 | 1.172 | 0.521 | 2.398 | NA | NA | NA |
| 13 | 1.060 | 0.078 | 1.900 | 0.944 | 0.937 | 1.812 | NA | NA | NA |
| 14 | 0.687 | -0.076 | 2.622 | 0.769 | 1.884 | 3.816 | NA | NA | NA |
| 15 | 0.610 | -0.199 | 2.769 | 0.612 | 0.057 | 2.222 | NA | NA | NA |
| 16 | 0.938 | 0.265 | 2.541 | 0.746 | 0.833 | 3.597 | NA | NA | NA |
| 17 | 0.497 | 6.045 | 2.589 | 0.146 | 33.542 | 8.326 | NA | NA | NA |
| 18 | 0.492 | 2.599 | 3.319 | 0.242 | 6.340 | 4.786 | NA | NA | NA |
| 19 | 0.728 | -0.257 | 2.983 | 0.613 | -0.111 | 3.612 | NA | NA | NA |
| 20 | 0.697 | 2.802 | 4.342 | 0.524 | 4.481 | 4.849 | NA | NA | NA |
| 21 | 1.201 | 0.789 | 1.951 | 0.583 | 2.568 | 5.326 | NA | NA | NA |
| 22 | 1.017 | -0.262 | 1.974 | 0.650 | -0.237 | 3.114 | NA | NA | NA |
| 23 | 1.059 | -1.702 | 1.232 | 1.436 | -0.940 | 1.557 | NA | NA | NA |
| 24 | 0.788 | -0.768 | 2.160 | 0.795 | -0.107 | 2.631 | NA | NA | NA |
| 25 | 1.110 | 0.924 | 2.686 | 1.335 | 0.778 | 2.880 | NA | NA | NA |
| 26 | 1.028 | -0.299 | 1.332 | 1.034 | -0.249 | 1.730 | NA | NA | NA |
| 27 | 1.309 | 0.994 | 2.401 | 1.491 | 1.082 | 2.463 | NA | NA | NA |
| 28 | 1.053 | 0.979 | 1.343 | 0.960 | 1.170 | 2.584 | NA | NA | NA |
| 29 | 1.897 | -0.349 | 1.711 | 2.094 | 0.128 | 1.690 | NA | NA | NA |
| 30 | 1.466 | 0.541 | 2.045 | 1.159 | 1.281 | 3.903 | NA | NA | NA |
| 31 | 1.544 | -1.197 | 1.207 | 1.319 | -0.950 | 1.797 | NA | NA | NA |
| 32 | 1.205 | -0.256 | 1.332 | 1.352 | -0.162 | 1.463 | NA | NA | NA |
| 33 | 0.749 | -0.839 | 1.037 | 0.632 | -0.187 | 1.127 | 0.580 | 0.827 | 1.325 |
| 34 | 1.360 | -1.116 | 0.602 | 1.226 | -1.300 | 0.410 | 1.472 | -1.577 | -0.068 |
| 35 | 0.903 | -1.306 | 1.050 | 1.114 | -1.213 | 0.393 | 0.922 | -1.724 | 0.195 |
| 36 | 1.092 | -0.391 | 0.963 | 1.170 | -0.170 | 0.940 | 1.278 | -0.144 | 1.166 |

## Explanation of the model

First, the restriction of the threshold parameters will be explained in detail. In each group, we have two group-specific threshold parameters (Group-specific threshold 1 and group-specific threshold 2) for each item. In the table below (Table S3), as an example, we presented group-specific thresholds from a scale that is consisting of 4 items. For the sake of simplicity, we presented group-specific thresholds only for one group and all of the presented thresholds have positive values.

The first step in the process of restriction of threshold parameters is to calculate the average group-specific threshold for each item in group 1 ($\beta_{1}$). The average group-specific threshold per item is equal to the sum of all group-specific thresholds of that item divided by the number of thresholds per item.

$$\tilde{\beta}_{kj}=\frac{\tilde{\beta}_{1kj}+\tilde{\beta}_{2kj}}{2}$$

In the case of the first item from the table below, we have group-specific threshold 1 for item 1 for group 1 $\tilde{\beta}_{111}$ = 2 and group-specific threshold 2 for item 1 for group 1 $\tilde{\beta}_{211}$ = 4. In order to calculate the average threshold for item 1 in group 1, $\tilde{\beta}_{11}$, we need to divide the sum of $\tilde{\beta}_{111}$ (group-specific threshold 1 for item 1 for group 1) and $\tilde{\beta}_{211}$ (group-specific threshold 2 for item 1 for group 1) with the number of thresholds (2).

Table S3 Rescaling of group-specific threshold parameters

| **Group 1** | **Item 1** | **Item 2** | **Item 3** | **Item 4** | **Mean (***µ***)** |
| --- | --- | --- | --- | --- | --- |
| Group-specific threshold 1 *β*˜ | 2 | 1 | 3 | 6 | 3 |
| Group-specific threshold 2 *β*˜ | 4 | 1 | 5 | 8 | 4.75 |
| Group 1 average threshold per item *β*˜ | 3 | 1 | 4 | 7 | 3.75 |
| Rescaled group-specific threshold 1 ∗*β*˜ | -1.75 | -2.75 | -0.75 | 2.25 | -3 |
| Rescaled group-specific threshold 2 ∗*β*˜ | 0.25 | -2.75 | 1.25 | 4.25 | 3 |
| Group 1 average rescaled threshold per item ∗*β*˜ | -0.75 | -2.75 | 0.25 | 3.25 | 0 |

In the previous step, we calculated $\tilde{\beta}_{k1}$, the average threshold for each item in Group 1. After that, we need to calculate the mean threshold in each group. We need to sum the average thresholds in a certain group and to divide that sum by the number of items K. In other words, we need to calculate $\mu_{\tilde{\beta}_{j}}$, the average of average thresholds $\tilde{\beta}_{kj}$ per group.

$$\mu_{\tilde{\beta}_{j}}=\frac{\sum_{k\in K} (\tilde{\beta}_{kj})}{K}$$

In the table above, we have data of four items from Group 1. Accordingly, we have four average thresholds in Group 1. In order to calculate the mean threshold in Group 1, we need to sum these four average thresholds of each item and do divide them by the number of items.

$$\mu_{\tilde{\beta}_{1}}=\frac{\tilde{\beta}_{11}+\tilde{\beta}_{21}+\tilde{\beta}_{31}+\tilde{\beta}_{41}}{4}=\frac{3+1+4+7}{4}=\frac{15}{4}=3.75$$

The mean threshold in Group 1 ($\mu_{\tilde{\beta}_{1}}$) is 3.75. Now, we can calculate the rescaled group-specific threshold parameters using this formula:

$$*\tilde{\xi}_{kj}=\tilde{\xi}_{kj}-\mu_{\tilde{\beta}_{j}}$$

Note that $\tilde{\xi}_{kj}$ consists of group-specific discrimination parameter ($\tilde{\alpha}_{kj}$) and two group-specific threshold parameters ($\tilde{\beta}_{1kj}$) and ($\tilde{\beta}_{2kj}$) for each item in each group in this study. As a consequence, the $*\tilde{\xi}_{kj}$ consist of rescaled group-specific discrimination parameter ($*\tilde{\alpha}_{kj}$), rescaled group-specific threshold parameter 1 ($*\tilde{\beta}_{1kj}$), and rescaled group-specific threshold parameter 2 ($*\tilde{\beta}_{2kj}$) for each item in each group. In this part of the text, we are focused on the threshold parameters, and the rescaling of the discrimination parameter will be explained later. Now, when all necessary terms and formulas are introduced, we will explain the last step in the process of rescaling group-specific threshold parameters. Based on the example introduced above, we will calculate the rescaled group-specific threshold parameters of four items presented in Table S3. The group-specific threshold 1, of item 1 in group 1, $*\tilde{\beta}_{111}$, is calculated by subtracting the mean threshold in Group 1, $\mu_{\tilde{\beta}_{1}}$, of group-specific threshold parameter 1 of item 1 in Group 1, $\tilde{\beta}_{111}$.

By applying this formula, we calculated the rescaled group-specific thresholds 1 and 2 for all four items in the table. At this point, we can see that the mean of rescaled group-specific threshold 1 parameters is -3, while the mean of rescaled group-specific threshold 2 parameters is 3, and their sum is equal to 0. As an illustration, we also calculated and presented the average rescaled threshold for each item in Group 1, where:

$$*\tilde{\beta}_{kj}=\frac{*\tilde{\beta}_{1kj}+*\tilde{\beta}_{2kj}}{2}$$

From Table S3, we can clearly see that the sum of the average rescaled thresholds in Group 1 is equal to 0. This implies that particular item parameters can have different thresholds, but the average threshold is the same in each group - 0. In other words, the assumption is that overall, the test has the same average threshold in each cohort. For example, some items in the Netherlands cohort can show relatively higher scores (lower thresholds), while some other items can show relatively lower scores (higher thresholds), but the average threshold is 0 within each cohort.

This kind of restriction in which the overall threshold in each group is equal to zero is used for identifying the model.

Similarly, the product of the discrimination parameters is restricted to one within each group. In the table below we presented group-specific discrimination parameters from a scale that is consisting of 4 items (Table S4) The first step in the process of restriction of discrimination parameters is to calculate the natural logarithm of each group-specific discrimination parameter in each group ($ln(\tilde{\alpha}_{kj})$).

After that, we calculated $\mu_{\tilde{\alpha}_{j}}$, an average of the logarithm of group-specific discrimination parameters, $ln(\tilde{\alpha}_{kj})$.

$$\mu_{\tilde{\alpha}_{j}}=\frac{\sum_{k\in K} (\tilde{\alpha}_{kj})}{K}$$

The K in this formula represents the number of items. As we can see in Table S4, $\mu_{\tilde{\alpha}_{j}}$, an average of the logarithm of group-specific discrimination parameters, is equal to 0.29.

In the next step, we calculated rescaled group-specific discrimination parameters using this formula:

$$*\tilde{\alpha}_{kj}=exp(ln(\tilde{\alpha}_{kj})-\mu_{\tilde{\alpha}_{j}})$$

As an example, the rescaled group-specific discrimination parameter of the first item is calculated in this way:

$$*\tilde{\alpha}_{11}=exp(ln(\tilde{\alpha}_{11})-\mu_{\tilde{\alpha}_{j}})=exp(0-.29)=exp(-.29)=.75$$

In the end, we can see that product of the rescaled group-specific discrimination parameters (0.75 * 0.93 * 0.38 * 3.74) is equal to 1. This kind of restriction in which the product of the discrimination parameters in each group is equal to 1 is necessary for identifying the model.

Table S4 Rescaling of group-specific discrimination parameters

| **Group 1** | **Item 1** | **Item 2** | **Item 3** | **Item 4** | **Mean (***µ***)** |
| --- | --- | --- | --- | --- | --- |
| Group-specific discrimination *α*˜*_k_*_1_ | 1 | 1.25 | 0.5 | 5 |  |
| The logarithm of group-specific discrimination ln(*α*˜*_k_*_1_) | 0 | 0.22 | -0.69 | 1.61 | 0.29 |
| ln(*α*˜*_k_*_1_) - *µ_α_*_˜_1 | -0.29 | -0.07 | -0.98 | 1.32 |  |
| Rescaled group-specific discrimination ∗*α*˜*_k_*_1_ | 0.75 | 0.93 | 0.38 | 3.74 |  |

To conclude, it is allowed for the particular item parameters to vary across cohorts, but the average threshold of the items is assumed to be equal (0) across cohorts. Particular item parameters can have different thresholds, but the average threshold is the same, 0 in each cohort.
